# Investigation The Role of Gender on The *HLA-DRB1* and -*DQB1* Association with Type 1 Diabetes Mellitus in Iranian Patients

**Published:** 2013-07-02

**Authors:** Arezou Sayad, Mohammad Taghi Akbari, Mohammad Pajouhi, Feridoon Mostafavi, Anooshirvan Kazemnejad, Mahdi Zamani

**Affiliations:** 1 Department of Neurogenetics, Iranian Centre of Neurological Research, Tehran University of Medical Sciences, Tehran, Iran; 2 Department of Medical Genetics, Faculty of Medical Science, Tarbiat Modares University, Tehran, Iran; 3 Department of Medical Genetics, School of Medicine, Tehran University of Medical Sciences, Tehran, Iran; 4 Department of Medical Science, Endocrine Research Center, Faculty of Medical Science, Tehran University of Medical Sciences, Tehran, Iran; 5 Department of Endocrinology, Children Medical Center, School of Medicine, Tehran University of Medical Sciences, Tehran, Iran; 6 Department of Biostatics, Faculty of Medical Science, Tarbiat Modares University, Tehran, Iran

**Keywords:** Type 1 Diabetes, *HLA-DRB1*, *HLA-DQB1*, Gender, Age at Onset

## Abstract

**Objective:**

Type 1 diabetes mellitus (T1D) is an autoimmune and multifactorial disorder. Subsequent analysis on human leukocyte antigen ( HLA) region shows that *HLA-DRB1* and -*DQB1* genes have the strongest association with T1D. In this study, for the first time, we investigated the influence of gender on the *HLA-DRB1* and -*DQB1* association with type 1 diabetes mellitus in Iranian patients in order to determine gender dependent HLA heterogeneity in Iranian T1D patients.

**Materials and Methods:**

In this case control study, the *HLA-DRB1* and -*DQB1* typing were performed on 105 Iranian T1D patients and 100 healthy controls. The data were evaluated by using Fisher exact test.

**Results:**

Our results indicate that DRB1*04:01, DQB1*03:02 alleles and DRB1*04:01- DQB1*03:02 haplotype were significantly more frequent in male T1D patients than females. Also, DRB1*03:01, DRB1*15:01, DQB1*06:01 alleles, DQB1*03:01/05:01 genotype, DRB1*03:01-DQB1*02:01 and DRB1*15:01-DQB1*06:01 haplotypes were significantly higher in female T1D group than males. Furthermore, our results showed that DRB1*04:01 and DQB1*03:02 alleles were significantly more frequent in male T1D patients 1-5 years old at onset than females with similar condition. The DRB1*03:01 allele and DRB1*03:01- DQB1*02:01 haplotype were significantly higher in female T1D patients 6-10 years old at onset than males with similar condition. The DRB1*15:01 allele and DRB1*15:01-DQB1*06:01 haplotype were significantly more frequent in female T1D patients 16-20 years old at onset than males with similar condition.

**Conclusion:**

Our findings suggest that gender has a significant influence on the distribution of *HLA-DR* and -DQ alleles, genotypes and haplotypes. Also, distribution of the *HLA-DRB1* and -*DQB1* alleles, genotypes and haplotypes vary based on the gender of T1D patients in different age at onset.

## Introduction

Type 1 diabetes (T1D) is a chronic autoimmune disease which is caused by the absence of insulin synthesis. It is characterized by the immunologically T lymphocyte mediated destruction of the insulin producing cells (beta cells) in the pancreatic islets of langerhans (1). The rate of the disease concordance in siblings (6%) and monozygotic twins (35-50%) shows that both genetic and environmental factors are contributed to the disease risk (2, 3). Therefore, the T1D is known as multifactorial disorder. The human leukocyte antigen (*HLA*) genes are located on the short arm of chromosome 6. The *HLA* genes are contributed in 50% of the genetic risk for T1D (4, 5). For the first time, the association of T1D and *HLA* class I alleles was studied in 1973 (6). Association of these alleles is secondary to the association of *HLA*-DR and -DQ alleles of the *HLA* class II which were investigated later (4). The *HLA*-DRB1 and -*DQB1* alleles are identified as the most important alleles which are associated with T1D in many ethnic groups (7-9). Due to strong linkage disequilibrium between the *HLA-DRB1* and -*DQB1* alleles, the highest genetic risk is conferred by their haplotypes in compared with particular alleles (10-16). The studies about the incidence of T1D showed that, Finland, Sardinia and Scandinavia have the highest worldwide prevalence of T1D (>35/100,000/year). T1D is less frequent in Asian populations (0.4-1.1/100000/ year) compared to Caucasians. The Japanese, Chinese and Koreans have a very low T1D incidence, approximately 1/100000/year (17). The high incidence of T1D in Scandinavians correlates with the high frequency of the DRB1*04:01- DQA1*03:01-DQB1*03:02 haplotype. The high incidence of T1D in Sardinians is due to both the high frequency of another susceptible haplotype (DRB1*03:01-DQA1*05:01-DQB1*02:01), and the low frequency of protective haplotype (DRB1*15:01-DQA1*01:02-DQB1*06:02) (18). In Japanese population, the absence of the DR3 alleles correlates with the low incidence of T1D. The share of DR3/4 genotype is also less in Koreans than in Caucasians (19). There are differences in the *HLA-DRB1* and -*DQB1* gene profiles according to the gender and age at onset (20, 21). Some studies reported that HLA-DRB1*03:01/09:01 was significantly more frequent in female T1D patient, whereas HLA-DRB1*03:01/04:01 had more frequency in male T1D patients (21, 22). Another study showed that DQ6.2 (DQA1* 01:02- DQB1*06:02) was more negatively associated with T1D in females than males (23). Finding whether gender influence on susceptibility to or protection against T1D, can help to better followup the T1D patients according to their sex category. Also, knowing the gender dependent *HLA* heterogeneity among T1D patients indicates influence of sex dependent factor, as the hormones, on the functional effects of the *HLA* molecules. The individuals carrying alleles which are associated with younger age at onset in each female or male group should take care under preventive treatment.

In the present study, we aimed to examine the influence of the gender on susceptible and protective alleles, genotypes and haplotypes of the **HLA*-DRB1* and -*DQB1* to find gender dependent *HLA* heterogeneity in Iranian T1D patients.

## Materials and Methods

### Subject and control groups

In this case control study, one hundred and five unrelated Iranian patients with T1D mellitus with a mean standard deviation age of 9.4 ± 4 years and an age range of 1-17 years (45% male and 55% female) were studied in the Department of Neurogenetics of the Iranian Center of Neurological Research. Patients with other type of diabetes, such as type 2 diabetes, maturity onset diabetes of youth and latent diabetes were not included in this study. Subjects were collected from the different hospitals located in Tehran and were diagnosed as having type1 diabetes mellitus by endocrinologist according to the WHO criteria. All patients as well as controls were of Iranian descent with mixed ethnic origin, and they were selected from different ethnic groups. One hundred healthy, ethnically and sex matched individuals with no personal or family history of autoimmune disorders (mean age of 36.2 ± 7.8 years and age range of 23-68 years) were selected as a control group in the same geographical area as patients (47% male and 53% female). All guardians were informed of the study and gave their written consent. The study was approved by the Ethnic Committee of the Tarbiat Modares University.

### HLA-DRB1 and -DQB1 typing and data collection

DNA was extracted from peripheral venous blood specimens using salting out method (24). The high resolution *HLA*-DRB typing was performed by Inno lipa DRB kit (Innogenetics NV, Belgium). It was conducted according to the manufacturer’s recommendations. HISTO TYPE SSP Low Resolution Kits (BAG Health care, Germany) were used for *HLA* Typing of *DQB1* as instructed by the manufacturer. Questionnaire information (such as sex, age at on set, ethnic, autoimmune or genetic history) of all patients and controls were collected. According to the results of *HLA*-DRB and -DQB typing, we determined susceptible and protective alleles, genotypes and haplotypes (25). Individuals were divided into two groups of male and female. Moreover, each male and female group was categorized into four groups based on age categories at onset (1-5, 6-10, 11-15 and 16-20). Distribution of susceptible and protective alleles, genotypes and haplotypes in each group was assessed.

### Statistical methods

The data were evaluated by using fisher’s exact test. Odds ratio (OR) was calculated. The p values less than 5% were considered statistically significant. All data were analyzed by SPSS 18.

## Results

All patients and controls were divided into two groups of male and female. Furthermore, each male and female group was categorized into four groups based on age categories at onset (1-5, 6-10, 11-15 and 16-20). Distribution of susceptible and protective alleles, genotype and haplotype in each group was determined.

### Distribution of susceptible and protective HLA-DRB1 and -*DQB1* alleles according to the gender of T1D

HLA-DRB1*04:01 allele had a 15.5 times higher risk of developing T1D in males than females. HLA-DQB1*03:02 allele (p=0.01; OR: 6.273) was significantly higher in male T1D patients compared to females. Female carriers of DQB1*06:01 allele had a 14 times higher risk of developing T1D than males. DRB1*03:01 (p=0.01; OR: 0.244) and HLA-DRB1* 15:01 (p=0.03) alleles were significantly more frequent in female T1D patients than males (Table 1).

**Table 1:** Distribution of susceptible and protective HLA-DRB1 and -DQB1 alleles according to the gender of T1D


Allele	Gender	TID	Control	P	OR(CL)

HLA-DRB1*0401	M	31	3	0.01	15.5 (1.813-132.543)
	F	2	3		
HLA-DRB1*0301	M	18	13	0.01	0.244 (0.079-0.751)
	F	34	6		
HLA-DRB1*1301	M	0	9	0.4	-
	F	1	7		
HLA-DRB1*1501	M	0	17	0.03	-
	F	4	11		
HLA-DQB1*0201	M	43	17	0.2	-
	F	31	21		
HLA-DQB1*0302	M	46	4	0.01	6.273 (1.507-26.106)
	F	11	6		
HLA-DQB1*0301	M	9	24	1	-
	F	15	40		
HLA-DQB1*0601	M	1	21	0.01	0.071 (0.007-0.682)
	F	6	9		


M; Male, F; Female, NS; Not significant and P; Value of Fisher’s exact test.

### Distribution of susceptible and protective HLA-DRB1 and -DQB1 genotypes were based on the gender of T1D

Frequency of susceptible and protective *HLA-DRB1* and -*DQB1* genotypes with respect to the gender of T1D patients and healthy controls are shown in table 2. The DQB1*03:01/05:01 (p=0.03) genotype was significantly higher in female T1D patients than males. Although HLA-DRB1* 03:01/04:01 and HLA-DQB1*02:01/03:02 genotypes were more frequent in male patients compared to females, but their differences were not significant.

### Distribution of susceptible and protective HLA-DRB1 and -DQB1 haplotypes according to the gender of T1D are as follows

HLA-DRB1*04:01-DQB1*03:02 (p: 0.03) haplotype was significantly more frequent in male T1D patients than female. HLA-DRB1* 03:01-DQB1*02:01 (p=0.02; OR: 0.241) and DRB1*15:01-DQB1*06:01 (p=0.01) haplotypes were significantly higher in female T1D patients than males. These results are summarized in table 3.

### Distribution of susceptible and protective HLA-DRB1 and -DQB1 alleles according to the gender of T1D in four age categories at onset groups are as follows

The HLA-DRB1*04:01 (p=0.03; OR: 17) and HLA-DQB1*03:02 (p=0.001) alleles were significantly more frequent in male T1D patients 1-5 years old at onset than females with similar condition. HLA-DRB1*03:01 (p=0.02; OR: 0.194) and HLA-DRB1*15:01 (p=0.03) alleles were significantly higher in female T1D patients 6-10 and 16- 20 years old at onset, respectively, than males with similar conditions (Table 4).

**Table 2:** Distribution of susceptible and protective HLA-DRB1 and -DQB1 genotypes according to the gender of T1D


	Gender	TID	Control	P	OR(CL)

HLA-DRB1*0301/0401	M	12	0	0.1	-
	F	1	1		
HLA-DQB1*0201/0302	M	11	0	0.2	
	F	3	1		
HLA-DQB1*0301/0501	M	0	12	0.03	-
	F	4	7		
HLA-DQB1*0301/0601	M	0	10	0.3	-
	F	1	4		


M; Male, F; Female, NS; Not significant and P; Value of Fisher’s exact test.

**Table 3:** Distribution of susceptible and protective HLA-DRB1 and -DQB1 haplotypes according to the gender of T1D


Haplotype	Gender	TID	Control	P	OR(CL)

DRB1*0401-DQB1*0302	M	31	0	0.03	-
	F	0	1		
DRB1*0301-DQB1*0201	M	18	11	0.02	0.241 (0.072-0.8)
	F	34	5		
DRB1*1501-DQB1*0601	M	0	15	0.01	-
	F	4	6		


M; Male, F; Female, NS; Not significant and P; Value of Fisher’s exact test.

### Distribution of susceptible and protective HLA-DRB1 and -DQB1 genotypes and haplotypes based on the gender of T1D in four age categories at onset groups are as follows

HLA-DQB1*02:01/03:02 genotype was more frequent in female patients compared to males, but the difference was not significant. HLA-DRB1* 03:01-DQB1*02:01 (p=0.007; OR: 0.121) and DRB1*15:01-DQB1*06:01 (p=0.01) haplotypes were significantly higher in female T1D patients 6-10 and 16-20 years old at onset groups, respectively, than males with similar conditions (Table 5).

**Table 4:** Distribution of susceptible and protective HLA-DRB1 and –DQB1 alleles according to the gender of T1D in four age categories at onset groups


Age categories at onset														
Allelle	Gender	1-5	P	OR(CL)	6-10	P	OR(CL)	11-15	P	OR(CL)	16-20	P	OR(CL)	Control

DRB1*0401	M(n:31)	17	0.035	17(12-223.1)	6	0.2	-	8	0.06	-	0	NS	-	3
	F(n:2)	1			1			0			0			3
DRB1*0301	M(n:18)	7	NS	-	5	0.02	0194(0.04-0.77)	10	NS	-	0	NS	-	12
	F(n:34)	8			15			7			0			7
DRB1*1501	M(n:0)	0	NS	-	0	NS	-	0	NS	-	0	0.03	-	17
	F(n:34)	0			0			0			4			11
DQB1*0302	M(n:46)	17	0.001	-	12	0.08	-	12	0.2	-	5	0.1	-	4
	F(n:11)	0			3			17			1			6
DQB1*0601	M(n:1)	0	NS	-	0	NS	-	0	0.1	-	1	0.056	-	21
	F(n:6)	0			0			2			4			9


M; Male, F; Female, NS; Not significant and P; Value of Fisher’s exact test.

**Table 5:** Distributions of susceptible and protective HLA-DRB1 and -DQB1 genotypes and haplotypes according to the gender of T1D in four age at onset groups


Age categories at onset
Genotype/haplotype	Gender	1-5	P	OR(CL)	6-10	P	OR(CL)	11-15	P	OR(CL)	16-20	P	OR(CL)	Control

Genotype DQB1*0301/0501	M(n:0)	0	NS	-	0	NS	-	0	0.4	-	0	0.07	-	12
	F(n:4)	0			0			4			3			7
Haplotype DRB1*0401-DQB1*0302	M(n:30)	13	0.07	-	8	0.1	-	10	0.9	-	0	NS	-	0
	F(n:1)	0			0			0			0			1
DRB1*0301-DQB1*0201	M(n:18)	4	0.1	-	4	0.007	0.121(0.02-0.55)	10	0.3	-	0	NS	-	11
	F(n:34)	8			15			4			0			5
DRB1*1501-DQB1*0601	M(n:0)	0	NS	-	0	NS	-	0	NS	-	0	0.01	-	15
	F(n:4)	0			0			0			4			6


M; Male, F; Female, NS; Not significant and P; Value of Fisher’s exact test.

## Discussion

In this study, we investigated the distribution of susceptible and protective HLA-*DRB1* and -*DQB1* alleles, genotypes and haplotypes based on the gender to find gender-dependent HLA heterogeneity in Iranian T1D patients.

Type 1 diabetes mellitus is a multifactorial and polygenic disease with concordance rate of 30- 50% in twins, indicating the significance of genetic factors (26, 27). Among various genes in HLA region, HLA-DRB and -DQB (HLA class II) contribute the strongest risk to T1D (28, 29). However, differences in HLA-*DRB1* and -*DQB1* haplotypes have been associated with T1D in different population. Exact mechanism of susceptibility to T1D still remains unclear.

In current study, it was found out that HLA-DRB1* 04:01 and DQB1*03:02 alleles and DRB1*04:01-DQB1*03:02 haplotype were significantly more frequent in male patients than females. Chan et al. already showed that frequency of DRB1*04 was significantly higher in male T1D patients than females (22). Tait et al. reported that DR3-/DR4+ genotype was higher in male patients than female group, which is consistent with our results (30 ). Another study observed that DRB1*03:01/04:01 genotype was mainly in male T1D group (22). We demonstrated that DRB1*03:01/04:01 genotype was more frequent in male patients than females, but it was not significant. Chen et al. reported DQB1*02:01 allele was higher in male T1D group than females (20), whereas DQB1*02:01 allele was not significantly more frequent in Iranian male T1D patients. Also, there was observed that HLA-DRB1*03:01, 15:01 and DQB1*06:01 alleles, DRB1*03:01/05:01 genotype, DRB1*03:01-DQB1*02:01 and DRB1*15:01-DQB1*06:01 haplotypes were significantly higher in female T1D patients than male. Similar to our data, most studies demonstrated DRB1*0301 allele was more frequent in female T1D patients than male (22, 30). In Korean population, it was observed that DRB1*03:01/09:01 was mainly in female T1D group (22), but as DRB1*03:01/09:01 genotype was rare among Iranian population, we did not obtain significant any association between DRB1*03:02/09:01 genotype and gender in T1D patients. A study in Sweden by Graham et al. 1999 found that DQB1*06:02 was more negatively associated with T1D in females than males, while we demonstrated that DQB1*06:01 was more frequent in female T1D group than males (23).

We observed significant differences in age categories at onset in susceptible and protective alleles, genotypes and haplotypes of *DRB1* and *DQB1* stratified by the associated gender. We showed a gender difference in T1D patients which were diagnosed in category of 1-5 years old at onset, with an excess of males in the DRB1*04:01 and DQB1*03:02 groups. Also, frequencies of DRB1*03:01 allele and DRB1* 03:01-DQB1*02:01 haplotype in female patients diagnosed in category of 6-10 years old were significantly more than male patients. Similar to our results, Tait et al. showed a gender difference in T1D patients diagnosed at age < 12 years with an excess of males in DR3-/ DR4+ group and females in DR3+/DR4- group (30). Chan et al. demonstrated that male T1D patients were significantly higher in the frequency of DRB1*03:01/04:01 than female with an inverse relation to the age at onset, while DRB1*03:01/09:01 was observed more frequent in female T1D patients than males with an inverse relation to the age at onset (22). Finding whether gender influence on susceptibility to or protection against T1D, can help to better flow up the T1D patients according their sex category. Also, knowing the gender dependent HLA heterogeneity among T1D patients indicates the influence of sex dependent factor, as the hormones, on the functional effects of the HLA molecules. The individuals carrying alleles which are associated with younger age at onset in each female or male group should take care under preventive treatment.

## Conclusion

We can conclude that there is gender dependent HLA genetic heterogeneity of type 1 diabetes in Iranian patients. Also, distribution of HLA-*DRB1* and -*DQB1* alleles, genotypes and haplotypes according to the gender are significantly different in diagnosed age at onset.
